# Immune checkpoint inhibitors-associated thrombosis in patients with lung cancer and melanoma: a study of the Spanish society of medical oncology (SEOM) thrombosis and cancer group

**DOI:** 10.1007/s12094-022-02860-5

**Published:** 2022-06-06

**Authors:** Manuel Sánchez Cánovas, David Fernández Garay, Laura Ortega Moran, Jaime Rubio Pérez, Carlos Miguel Guirao Rubio, Miriam Lobo de Mena, Berta Obispo Portero, Jesús Brenes Castro, Yolanda Lage, Diego Cacho Lavin, Ana Belen Rupérez Blanco, Ana Manuela Martín Fernández de Soignie, Jonatan Zacarías Benoit Perejón, Laura Jiménez Colomo, Noel Blaya Boluda, Javier Bosque Moreno, Teresa Quintanar Verduguez, Carmen Rosa Garrido, Roberto Martín Huertas, Carme Font i Puig, Andrés Jesús Muñoz Martín

**Affiliations:** 1 Spanish Society of Medical Oncology (SEOM) Thrombosis and Cancer Group, Madrid, Spain; 2 Hematology and Medical Oncology Department, Hospital Universitario José María Morales Meseguer, Murcia, Spain; 3 Medical Oncology Department, Complejo Hospitalario de Jaén, Jaén, Spain; 4 Medical Oncology Department, Hospital General Universitario Gregorio Marañón, Instituto de Investigación Sanitaria Gregorio Marañón, Madrid, Spain; 5 Medical Oncology Department, Fundación Jiménez Díaz, Madrid, Spain; 6 Medical Oncology Department, Hospital General Universitario de Elche, Elche, Spain; 7 Medical Oncology Department, Hospital General Universitario de Valencia, Valencia, Spain; 8 Medical Oncology Department, Hospital Universitario Infanta Leonor, Madrid, Spain; 9 Instituto Catalán de Oncología, L’Hospitalet de Llobregat, Barcelona, Spain; 10 Medical Oncology Department, Hospital Universitario Ramón y Cajal, Madrid, Spain; 11 Medical Oncology Department, Hospital Universitario Marqués de Valdecilla, Santander, Spain; 12 Medical Oncology Department, Complejo Hospitalario de Toledo, Toledo, Spain; 13 Medical Oncology Department, Hospital Universitario de Fuenlabrada, Fuenlabrada, Spain; 14 Unidad de Investigación, Hospital Universitario de Jaén, FIBAO, Jaén, Spain; 15 Medical Oncology Department, Hospital Clínic de Barcelona, Barcelona, Spain

**Keywords:** Immune Checkpoint Inhibitors, Cancer related thrombosis, Lung cancer, Melanoma

## Abstract

**Purpose:**

Immune Checkpoint Inhibitors (ICI) can be associated with thrombotic events, both venous and arterial (VTE/AT). However, there is a paucity of information regarding patients in routine clinical practice.

**Methods/patients:**

Retrospective, multicenter study promoted by the Thrombosis and Cancer Section of the Spanish Society of Medical Oncology (SEOM). Patients with melanoma and lung cancer who initiated ICI between 01/01/2015 and 31/12/2019 were recruited. Minimum follow-up was 6 months (unless it was not possible because of death). The primary objective was to calculate the incidence of ICI-associated VTE/AT and the secondary objectives included to analyze its impact on survival and to identify predictor variables for VTE/AT.

**Results:**

665 patients with lung cancer were enrolled. The incidence of VTE/AT during follow-up was 8.4%. Median overall survival (OS) was lower in the VTE/AT group (12 months 95% CI 4.84–19.16 vs. 19 months 95% CI 16.11–21.9; *p* = 0.0049). Neutrophil/lymphocyte ratio (NLR) and anemia upon initiation of IT, as well as a history of thrombosis between cancer diagnosis and the start of ICI, were predictive variables for developing of VTE/AT (*p* < 0.05). 291 patients with melanoma were enrolled. There was a 5.8% incidence rate of VTE/AT during follow-up. Median OS was lower in the VTE/AT group (10 months 95% CI 0.0–20.27 vs. 29 months 95% CI 19.58–36.42; *p* = 0.034). NLR and lactate dehydrogenase (LDH) at the beginning of ICI were predictor variables for VTE/AT (*p* < 0.05).

**Conclusions:**

ICI increases the risk of VTE/AT in patients with lung cancer and melanoma, which impact OS.

## Introduction

Per se, cancer patients display certain clinical factors that can promote the development of thrombosis. However, apart from these elements, reasonable doubt remains as to whether Immune Checkpoint Inhibitors (ICI) increases thrombotic risk.

ICI has represented a highly significant change in the management of certain oncological patient profiles. Leading clinical guidelines on immunotoxicity report enteritis, endocrinopathies, neurological disorders, skin alterations, and more. The latest update published by the American Society of Medical Oncology (ASCO) recognizes thrombotic events as one such possible adverse effects associated with ICI [1].

Similarly, the literature also contains several case reports speculating on possible causal relationships between the use of ICI and the development of venous or arterial thromboembolic disease, with pembrolizumab, nivolumab, or ipilimumab [2–4].

Molecular studies yield additional answers. T-cell activation, bolstered by ICI, is known to be capable of inducing cytokine release, especially interferon gamma, which leads to increased tissue factor expression by circulating monocytes/macrophages, provoking a state of hypercoagulability [5, 6]. Furthermore, other studies suggest that PD1 blockade can induce the formation of atherosclerotic plaques, which would account for the appearance of arterial phenomena [7, 8]. Likewise, ICI can trigger alterations in the fibrinolysis system that induce greater propensity toward pro-thrombotic states, which seems more likely in individuals with tumors that express PD1 ligand (PDL1) in greater quantities, as well as in the early stages of ICI [9].

With all this information in hand, this study was designed to probe the relationship between thrombosis and ICI in patients in routine clinical practice.

## Materials and methods

This is a study sponsored by the Thrombosis and Cancer Section of the Spanish Society of Medical Oncology (SEOM). It is a retrospective, multicenter study (13 centers participated). Data from patients with melanoma and lung cancer who initiated ICI between 01/01/2015 and 31/12/2019 were collected. Selection was independent of tumor stage, type of ICI, or intentionality of treatment. Participants had to have a minimum follow-up of 6 months (unless it was not possible because of death).

Two independent cohorts were established, one containing cases of lung cancer and the other, melanoma. For both cohorts, the primary objective was to calculate the incidence of thrombosis associated with ICI.

Two secondary objectives were defined. The first was to examine the impact of thrombosis on survival among subjects treated with ICI, using the Kaplan–Meier method and log-rank test. The second was to find predictor variables for the development of thrombosis in individuals treated with ICI using multivariate analysis techniques (Cox Proportional-Hazards Model).

Prior to its implementation, the study was submitted to the Ethics Committee of each participating centers and obtained the corresponding approval. The processing, communication, and transfer of all personal data complied with the provisions of Organic Law 15/1999, dated December 13, 1999, regarding the protection of personal data and of Organic Law 3/2018, dated December 5, 2018, since its entry into force.

## Results

### Lung cancer

A total of 665 patients were recruited; baseline characteristics are displayed in Table 1. This cohort had a median age of 64 years; most were male (69.6%). Functional status in this group was good (92.9% with ECOG 0–1). Most had adenocarcinoma-like histology (57.7%) and disseminated oncological disease (91.2%, stage IV) when they started ICI.

**Table 1:** Baseline characteristics of the sample of lung cancer patients

Parameter	Subparameter	*n* = 665
Gender	Male	69.6% (*n* = 463)
	Female	30.4% (*n* = 202)
BMI	< 18.5 kg/m^2^	5.7% (*n* = 38)
	18.5–24.9 kg/m^2^	42.9% (*n* = 285)
	25–29.9 kg/m^2^	35.6% (*n* = 237)
	> 30 kg/m^2^	15.8% (*n* = 105)
Smoking status	Never smoked	9.6% (*n* = 64)
	Active smoker	43.0% (*n* = 286)
	Ex-smoker	47.4% (*n* = 315)
Medical history unrelated to the current lung cancer	HBP	43.0% (*n* = 286)
	DM	19.7% (*n* = 131)
	DLP	36.7% (*n* = 244)
	Thrombophilia	0.8% (*n* = 5)
	Heart stroke	6.0% (*n* = 40)
	Chronic CV disease	14.7% (*n* = 98)
	Peripheral vascular disease	8.3% (*n* = 55)
	COPD	22.4% (*n* = 149)
	Autoimmune disease	4.7% (*n* = 31)
	Liver disease	5.4% (*n* = 36)
	CKD	4.4% (*n* = 29)
	CVD	4.5% (*n* = 30)
	Other previous malignancies	14.0% (*n* = 93)
	VTE/ AT (during the 6 months preceding the diagnosis of cancer)	5.1% (*n* = 34)
	Concomitant hormonal therapy	1.2% (*n* = 8)
	Concomitant EPO	0.3% (*n* = 2)
	PICC or port-a-cath carrier	13.7% (*n* = 91)
Tumor stage at ICI initiation	Stages I**–**III	8.8% (*n* = 59)
	Stage IV	91.2% (*n* = 606)
Histology	Adenocarcinoma	57.7% (*n* = 384)
	Epidermoid	32.0% (*n* = 213)
	Others	10.3% (*n* = 68)
Mutational study	PDL1 ≥ 1%	68.1% (*n* = 453)
	ALK translocation positive	0.2% (*n* = 1)
	Mutated EGFR	0.5% (*n* = 3)
	Mutated ROS1	0.3% (*n* = 2)
	Mutated BRAF	0.5% (*n* = 3)
PDL1 expression	Unknown/ Not available	31.9% (*n* = 212)
	< 1%	4.7% (*n* = 31)
	1**–**49%	16.7% (*n* = 111)
	> 50%	46.8% (*n* = 311)
ECOG at start of ICI	0**–**1	92.9% (*n* = 618)
	2**–**3	7.1% (*n* = 47)
Treatment modality in which ICI was used	First-line metastatic disease	47.1% (*n* = 313)
	Second-line metastatic disease	36.1% (*n* = 240)
	Third or subsequent line of metastatic disease	8.0% (*n* = 53)
	Adjuvant/ Neoadjuvant	8.9% (*n* = 59)
Treatment regimen	Pembrolizumab in monotherapy	42.7% (*n* = 284)
	Nivolumab in monotherapy	21.1% (*n* = 140)
	Atezolizumab in monotherapy	15.8% (*n* = 105)
	Durvalumab in monotherapy	6.5% (*n* = 43)
	Pembrolizumab plus chemotherapy	6.0% (*n* = 40)
	Others	7.8% (*n* = 53)

*AT* arterial thrombosis, *CKD* chronic kidney disease, *COPD* chronic obstructive pulmonary disease, *CV* cardiovascular disease, *CVD* cerebrovascular disease, *DLP* dyslipemia, *DM* diabetes mellitus, *EPO* erythropoietin, *HBP* high blood pressure, *ICI* immune checkpoint inhibitors, *PICC* pheriperally inserted central catheter, *VTE* venous thromboembolism

ICI was mainly used in the context of first- (47.1%) or second-line (36.1%) for advanced disease. Almost half (42.7%) of the present cohort received pembrolizumab in monotherapy as an antineoplastic treatment modality. Among those patients in whom the determination of PDL1 could be performed (*n* = 453), the majority (68.6%) had an expression greater than 50%.

Regarding thrombotic history, 5.1% of the subjects had a previous history of VTE/AT that had been diagnosed prior to 6 months before diagnosis of lung cancer. In the interval between cancer diagnosis and date of ICI initiation, 8.7% the cases had VTE/AT.

The incidence of VTE/AT associated with ICI during follow-up (median 14 months) was 8.4% (95% confidence interval [CI] 6.23–10.6) (*n* = 56). A median of 5 ICI cycles administered at the time of diagnosis of VTE/AT (interquartile range [IQR] 1.25–11). At the time of VTE/AT diagnosis, 25.86% of the patients in the cohort were receiving anticoagulant therapy (13.8% at prophylactic doses, 12.06% at therapeutic doses). With respect to the characteristics of the VTE/AT episodes (Table 2), the most common form of thrombosis was pulmonary embolism (PE) (46.4%). As for arterial events, 7% of patients had suffered a cerebral stroke, while 5.4% had suffered heart stroke.

**Table 2:** Characteristics of VTE/ AT episodes in patients with lung cancer

Parameter	Subparameter	*n* = 56
Type VTE/AT	PE	46.4% (*n* = 26)
	DVT	17.9% (*n* = 10)
	Other forms of VTE: visceral, associated with catheter…	17.9% (*n* = 10)
	Cerebral stroke	7.0% (*n* = 4)
	Heart stroke	5.4% (*n* = 3)
	Other forms of AT	5.4% (*n* = 3)
Tumor reevaluation at diagnosis of VTE/AT	Complete response	5.4% (*n* = 3)
	Partial response	17.9% (*n* = 10)
	Stable disease	12.5% (*n* = 7)
	Progression	39.3% (*n* = 22)
	Not reevaluated	25.0% (*n* = 14)
The time of the VTE/AT	In the first 3 month post ICI initiation	44.6% (*n* = 25)
	Between 3 and 6 month post ICI initiation	21.4% (*n* = 12)
	More than 6 months after ICI initiation	33.9% (*n* = 19)
VTE/AT presentation	Incidental	33.9% (*n* = 19)
	Symptomatic	66.1% (*n* = 37)
Setting of VTE/AT diagnosis	Outpatient	82.1% (*n* = 46)
	In-patient	17.9% (*n* = 10)
Setting of VTE/AT management	Outpatient	48.2% (*n* = 27)
	In-patient	51.8% (*n* = 29)

*AT* arterial thrombosis, *DVT* deep vein thrombosis, *ICI* immune checkpoint inhibitors, *PE* pulmonary embolism, *VTE* venous thromboembolism

In those cases in which reevaluation was performed coinciding with the diagnosis of thrombosis, oncological disease was found to be progressing in more than half of the participants (52.27%). Approximately half of the events (44.66%) occurred within the first 3 months of initiating ICI. Two thirds (66.1%) of the thromboses were symptomatic. Initial management was undertaken in hospital in 51.8% of the cohort, although most subjects (82.1%) were diagnosed in an outpatient setting.

After VTE/AT, 41.1% of the patients in the cohort had their ICI suspended. Suspension was marked, because thrombosis was associated with progression. In only one case was ICI discontinuation motivated by the severity of the thrombotic event. Regarding post-VTE/AT complications, within the follow-up period there was the same percentage of rethrombosis and bleeding (8.9%).

The multivariate analysis (Table 3) revealed a statistically significant association of three variables with the risk of VTE/AT: hemoglobin levels < 10.9 g/dL at the start of ICI, neutrophil/lymphocyte ratio (NLR) > 4.55 at the beginning of ICI, and diagnosis of thrombosis during the interval between cancer diagnosis and initiation of ICI.

**Table 3:** Variables significantly correlated with thrombosis in lung cancer patients receiving ICI

	Univariate analysis			Multivariate analysis		
	HR	95% CI	*p* value	HR	95% CI	*p* value
Hemoglobin at initiation of ICI (cutoff < 10.9 g/dl)	2.35	1.32–4.16	0.004	2.05	1.14–3.69	0.008
Neutrophil/ lymphocyte ratio at initiation of ICI (cutoff > 4.55)	2.30	1.36–3.9	0.002	2.14	1.24–3.67	0.010
VTE/ AT between diagnosis of cancer and initiation of ICI	2.30	1.12–4.68	0.023	2.45	1.2–5.01	0.010

*AT* arterial thrombosis, *CI* confidence interval, *HR* hazard ratio, *ICI* immune checkpoint inhibitors, *VTE* venous thromboembolism

**Fig. 1:**
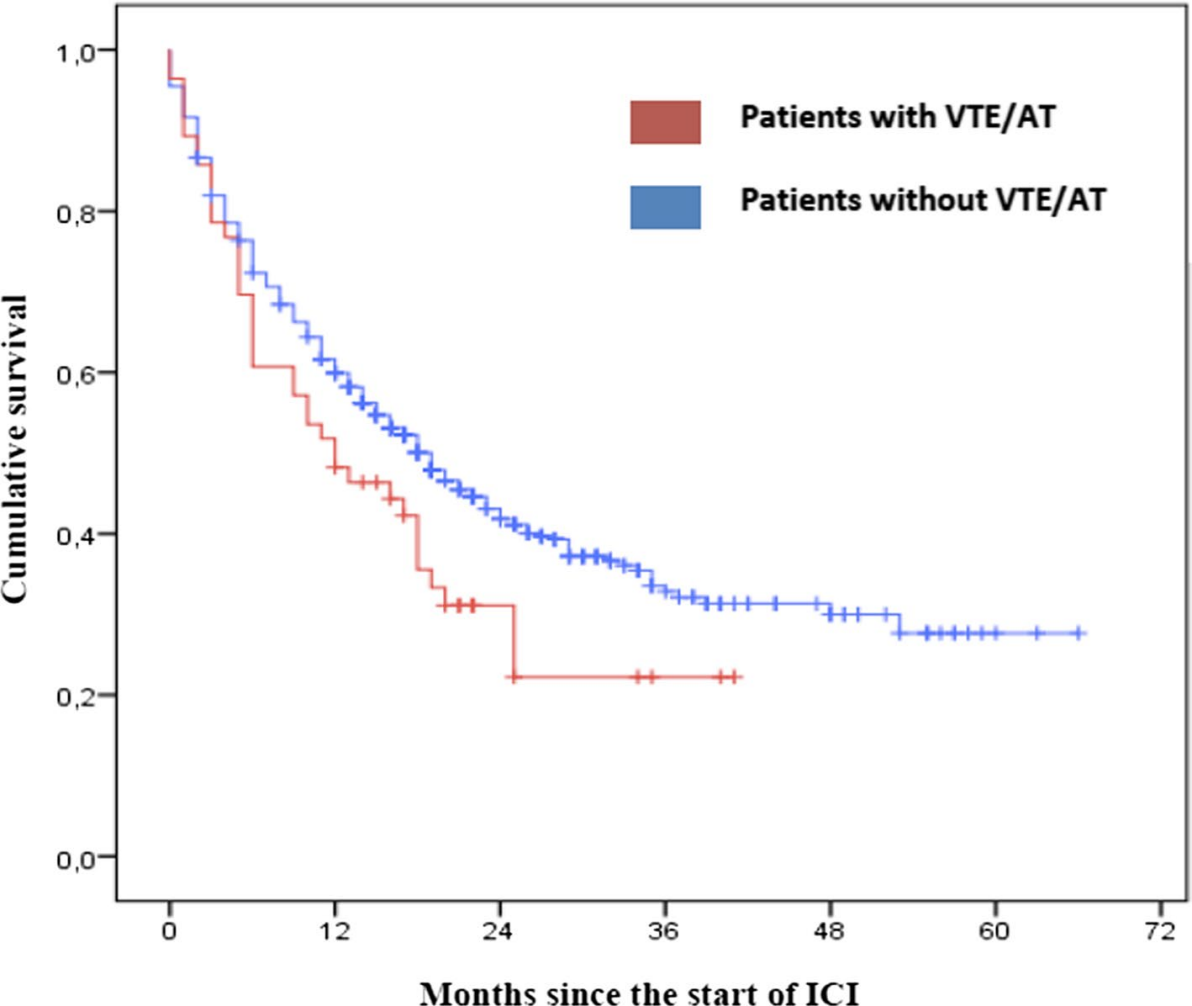
Kaplan–Meier curve comparing OS of lung cancer patients treated with ICI who developed VTE/AT vs. those who did not

Survival analysis (Fig. 1) revealed that median OS was lower in the group with VTE/AT (12 months, 95% CI 4.84–19.16) than in the group without VTE/AT (19 months 95% CI 16.11–21.9); the differences were statistically significant (*p* = 0.0049).

### Melanoma

A total of 291 patients were recruited. Their baseline characteristics can be seen in Table 4. This cohort had a median age of 62 years, with a similar proportion of men to women (52.6% and 47.4%, respectively). Their functional status was good (92.9% ECOG 0–1). At the start of ICI, 82.5% of the subjects had stage IV disease. From a molecular point of view, 29.5% of patients were BRAF mutated. PD-L1 had been determined in only 8.93% of the patients in the cohort with most (84.6%) being PD-L1 negative (< 1%).

**Table 4:** Baseline characteristics of the sample of patients with melanoma

Parameter	Subparameter	*n* = 291
Gender	Male	52.6% (*n* = 153)
	Female	47.4% (*n* = 138)
BMI	< 18.5 kg/m^2^	3.4% (*n* = 10)
	18.5–24.9 kg/m^2^	30.6% (*n* = 89)
	25–29.9 kg/m^2^	36.8% (*n* = 107)
	> 30 kg/m^2^	29.2% (*n* = 85)
Smoking	Never smoked	66.7% (*n* = 194)
	Active smoker	15.8% (*n* = 46)
	Former smoker	17.5% (*n* = 51)
Medical history unrelated to current melanoma	HBP	40.2% (*n* = 117)
	DM	21.0% (*n* = 61)
	DLP	24.7% (*n* = 72)
	Thrombophilia	0.7% (*n* = 2)
	Heart stroke	2.1% (*n* = 6)
	Chronic CV disease	10.0% (*n* = 29)
	Peripheral vascular disease	2.4% (*n* = 7)
	COPD	5.2% (*n* = 15)
	Autoimmune disease	4.5% (*n* = 13)
	Liver disease	1.0% (*n* = 3)
	CKD	3.1% (*n* = 9)
	CVD	3.1% (*n* = 9)
	Other previous malignancies	9.3% (*n* = 27)
	VTE/AT (during the 6 months preceding the diagnosis of cancer)	3.4% (*n* = 10)
	Concomitant hormonal therapy	1.0% (*n* = 3)
	Concomitant EPO	0.3% (*n* = 1)
	PICC or port-a-cath carrier	10.3% (*n* = 30)
Tumor stage at ICI initiation	Stages I**–**III	4.5% (*n* = 13)
	Stage IV	97.2% (*n* = 278)
Mutational status	PDL1 ≥ 1%	1.4% (*n* = 4)
	Mutated BRAF	29.6% (*n* = 86)
ECOG at ICI initiation	0**–**1	92.9% (*n* = 278)
	2**–**3	7.1% (*n* = 13)
Treatment modality in which the ICI was used	First-line metastatic disease	62.5% (*n* = 182)
	Second-line metastatic disease	17.5% (*n* = 51)
	Third or subsequent line metastatic disease	2.4% (*n* = 7)
	Adjuvant/Neoadjuvant	17.5% (*n* = 51)
Treatment regimen	Nivolumab in monotherapy	40.2% (*n* = 117)
	Pembrolizumab in monotherapy	36.1% (*n* = 105)
	Nivolumab + ipilimumab	7.2% (*n* = 21)
	Others	16.5% (*n* = 48)

*AT* arterial thrombosis, *CKD* chronic kidney disease, *COPD* chronic obstructive pulmonary disease, *CV* cardiovascular disease, *CVD* cerebrovascular disease, *DLP* dyslipemia, *DM* diabetes mellitus, *EPO* erythropoietin, *HBP* high blood pressure, *ICI* immune checkpoint inhibitors, *PICC* pheriperally inserted central catheter, *VTE* venous thromboembolism

ICI was mainly used in first-line setting (62.5%); the percentages of ICI in second-line/adjuvant setting were the same (17.5%). The most commonly used treatment regimens consisted of nivolumab (40.2%) and pembrolizumab (36.1%) in monotherapy.

In terms of thrombotic history, 3.4% of patients had a previous history of VTE/AT; these events had been diagnosed within 6 months prior to the diagnosis of melanoma. During the time period between cancer diagnosis and start of onset of ICI, 1.4% of the participants developed VTE/AT.

The incidence of VTE/AT associated with ICI during follow-up (median 17 months) was 5.8% (95% CI 3.34–9.18) (*n* = 17). A median of 8 ICI cycles had been administered at diagnosis of VTE/AT (interquartile range 2–11.75). At the time of VTE/AT diagnosis, 11.8% of patients in the cohort were receiving anticoagulant therapy (5.9% at prophylactic doses, 5.9% at therapeutic doses).

As for the characteristics of the VTE/AT episodes (Table 5), PE was the most common form of thrombosis (52.9%). With respect to arterial events, 5.9% of the cohort suffered a cerebral stroke, while 5.9% had an heart stroke. In those cases in which reevaluation was performed coinciding with the diagnosis of thrombosis, more than half (46.15%) were found to be in progression.

**Table 5:** Characteristics of VTE/ AT episodes in patients with melanoma

Parameter	Subparameter	*n* = 17
Type of VTE/AT	PE	52.9% (*n* = 9)
	DVT	17.6% (*n* = 3)
	Other forms of VTE: visceral, associated with catheter…	11.8% (*n* = 2)
	Cerebral stroke	5.9% (*n* = 1)
	Heart stroke	5.9% (*n* = 1)
	Other forms of AT	5.9% (*n* = 1)
Tumor reevaluation at diagnosis of VTE/AT	Complete response	23.5% (*n* = 4)
	Partial response	5.9% (*n* = 1)
	Stable disease	11.8% (*n* = 2)
	Progression	35.3% (*n* = 6)
	Not reevaluated	23.5% (*n* = 4)
The time of the VTE/AT	In the first 3 month post ICI initiation	29.4% (*n* = 5)
	Between 3 and 6 month post ICI initiation	29.4% (*n* = 5)
	More than 6 months after ICI initiation	41.2% (*n* = 7)
VTE/AT presentation	Incidental	41.2% (*n* = 7)
	Symptomatic	58. 8% (*n* = 10)
Setting of VTE/AT diagnosis	Outpatient	64.7% (*n* = 11)
	In-patient	35.3% (*n* = 6)
Setting of VTE/AT management	Outpatient	41.2% (*n* = 7)
	In-patient	58.8% (*n* = 10)

*AT* arterial thrombosis, *DVT* deep vein thrombosis, *ICI* immune checkpoint inhibitors, *PE* pulmonary embolism, *VTE* venous thromboembolism

Approximately half of the events (41.2%) occurred after the first 6 months of ICI. More than half (58.8%) of the thromboses were symptomatic. Initial management was inpatient in 58.8% of the cohort, although most patients (64.7%) were diagnosed in an outpatient setting.

ICI was discontinued after VTE/AT in 64.7% of the subjects. Suspension was indicated because of progression in 54.5%, while in 45.5%, ICI was interrupted due to the severity of the thrombotic episode. Apropos post-VTE/AT complications, the same percentage of rethrombosis and bleeding (11.8%) occurred during follow-up.

**Fig. 2:**
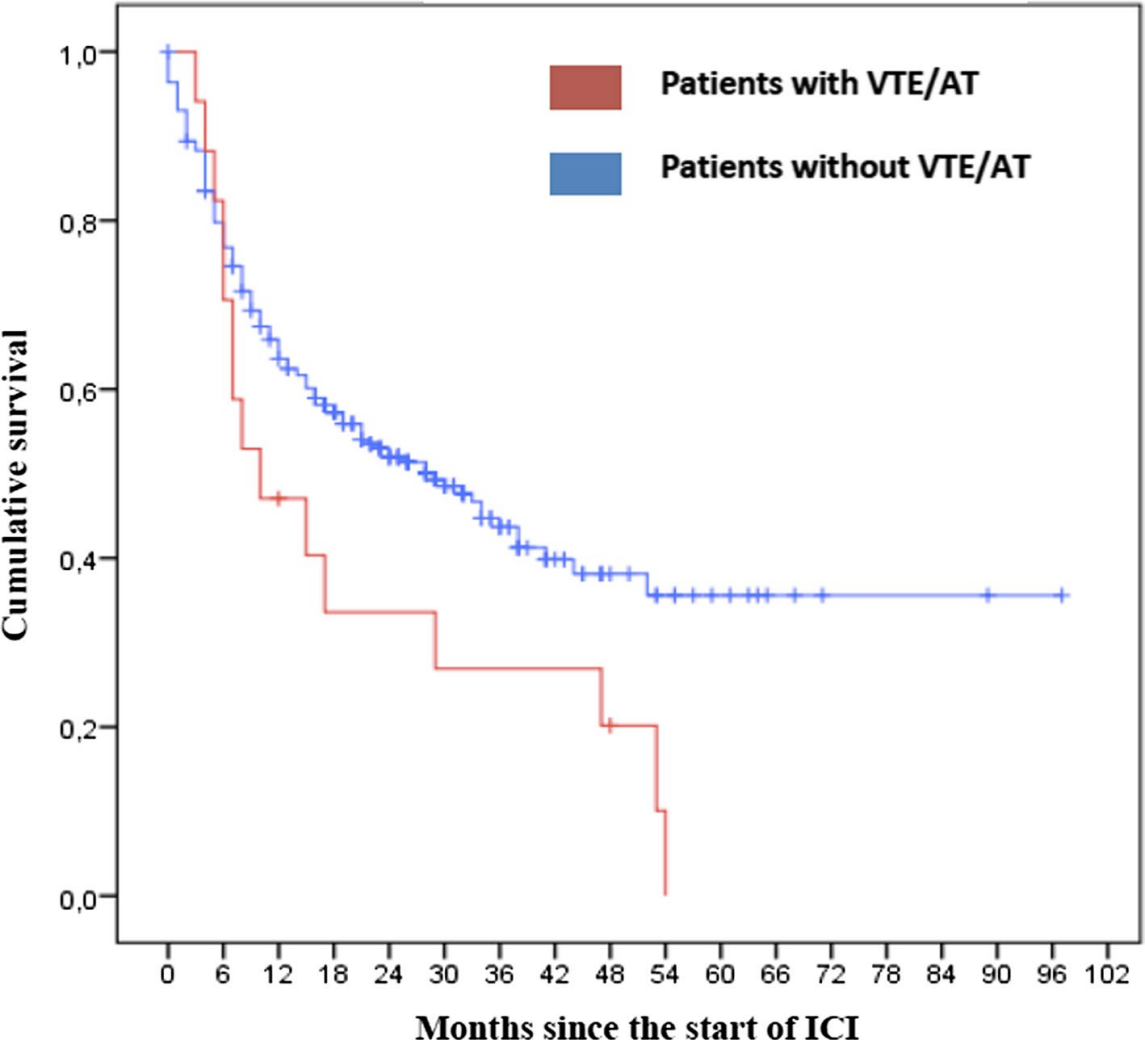
Kaplan–Meier curve comparing OS of melanoma cancer patients treated with ICI who developed VTE/AT vs. those who did not

Multivariate analysis (Table 6) revealed a statistically significant association of two variables with the risk of VTE/AT. These variables were: LDH > 198 U/L and NLR > 3.01%. Survival analysis (Fig. 2) evidenced that median OS was lower in the group with VTE/AT (10 months 95% CI 0.0–20.27) in contrast to the group without VTE/AT (29 months 95% CI 19.58–36.42); differences were statistically significant (*p* = 0.034).

**Table 6:** Variables significantly correlated with thrombosis in melanoma patients receiving ICI

	Univariate analysis			Multivariate analysis		
	HR	95% CI	*p* value	HR	95% CI	*p* value
LDH at initiation of ICI (cutoff ≥ 198)	5.50	1.32–4.16	0.025	4.51	1.01–20.24	0.049
Neutrophil/lymphocyte ratio at initiation of ICI (cutoff ≥ 3.01)	4.34	1.36–3.9	0.003	3.65	1.25–10.62	0.018

*AT* arterial thrombosis, *CI* confidence interval, HR hazard ratio, *ICI* immune checkpoint inhibitors, *VTE* venous thromboembolism

## Discussion

Medical oncology is currently witnessing an era in which the use of ICI is spreading to more and more neoplasia, as well as to a greater number of indications within each of these diseases. We are able to identify toxicities that have not been reported in pivotal clinical trials. ICI-associated VTE/AT is one such toxicity. The purpose of this research project was to provide more information within this area of medical oncology by dint of patients from routine clinical practice. The authors contend that there are three fundamental aspects that must be discussed: the incidence of IT-associated VTE/AT, its impact on OS in the oncological patient, and the identification of factors that can predict this type of complication.

As concerns incidence, Solinas et al. [10] published a systematic review in which they sought out the incidence of VTE/AT in individuals treated with ICI, within the framework of the leading clinical studies conducted with this type of therapy. They established that the incidence was low: 2.7% (95% CI 1.8–4) and 1.1% (95% CI 0.5–2.1) for VTE and AT, respectively. However, studies published subsequently and including cases in routine clinical practice reported higher figures.

For example, Wang et al. [11] performed a bibliographic review of VTE/AT associated with ICI. The patient profile most often associated with this type of complication was male, with stage IV disease, and with a primary lung, kidney, or melanoma. The data is comparable with our series, inasmuch as the cohorts comprise subjects with lung cancer and melanoma; moreover, in both groups, males with stage IV disease are the majority.

According to Wang et al. [11] the cumulative incidence of VTE was 5–8% and 10% at 6 months and 12 months from the start of ICI, respectively. As for arterial events, the incidence was 1–5% at 12 months from ICI initiation. While venous and arterial events were analyzed jointly in our series, this did not generate a significant variation in the percentages reported (8.7% in the lung cancer group and 5.7% in the melanoma group).

Other authors have also independently studied the incidence of venous and arterial events. Moik et al. [12] published a study of cases treated at the University Hospital of Vienna. All patients treated with ICI between 2015 and 2018 were enrolled in their research piece. This work included individuals with different cancers, in contrast to this series. Nonetheless, the most prevalent ones were melanoma (30.4%) and non-small cell lung cancer (24.1%). After a median follow-up of 8.5 months, the incidence of VTE was 12.9% and that of AT was 1.8%. In contrast, the results of the study by Ando Y et al. [13] are striking in that they detected an incidence rate of VTE that was lower (4.1%) than that of AT (4.9%).

The literature also includes other publications in which the scope of study has been limited to venous events. One example is the work carried out by Kewan et al. [14], who performed a retrospective, multicenter study that enrolled a total of 552 patients with stage IV cancer treated with ICI. The incidence of VTE was 10.5%. Of similar note in this field is the study conducted by Gong et al. [15] that retrospectively analyzed a total of 2,854 subjects in a single-center study. They established that the risk of VTE increased as the time of exposure to IT increased; thus, at 6 months, they found a 7.4% risk and a 13.8% risk at 12 months.

Deschênes-Simard et al. [16] performed a retrospective, multicenter, cohort study involving 593 patients with non-small cell lung cancer from three centers in Canada and France. The cumulative incidence of VTE was 14.8%, which was almost twofold the rate reported in the lung cancer cohort in our series (8.7%).

Similarly, Sussman et al. [17] conducted a retrospective, cohort study of melanoma patients who received ICI at the Cleveland Clinic. The sample size (*n* = 228) was slightly smaller than our melanoma cohort (*n* = 291). While there were commonalities, such as a majority of stage IV disease, the cumulative incidence of VTE post IT initiation was 9.3% at 6 months and 16% at 12 months, higher than the rates we report (5.7%).

Finally, in this section of the discussion dedicated to discussing incidence, the work carried out by Gutierrez-Sainz et al. [18] is worth noting. Theirs was a single-center, retrospective study. They recruited a total of 229 patients. Like some of the previously mentioned authors, they studied venous events exclusively and described an incidence of 7%, occurring more frequently in patients with lung cancer and melanoma. These figures are closer to the ones we report for our series, which is interesting, given that this study was conducted at a Spanish center and in Spanish patients, as are ours.

All the data reflected in the previous paragraphs, together with those of our series, only confirm what the review by Goel et al. [19] concluded: there is a reasonable incidence of VTE/AT among individuals receiving IT, despite the fact that the figures do vary.

The second aspect to be addressed in this discussion is the impact of ICI-associated VTE/AT on survival in this population. It is well known that thrombosis negatively affects the prognosis of cancer patients and, in fact, it is reported as the second leading cause of death among people afflicted with cáncer [20].

The data from this series indicate that for people with either melanoma or lung treated with ICI, the development of VTE/AT is statistically significantly associated with worse survival. In fact, among those patients in whom it was possible to reevaluate the underlying oncological disease, most were found to be in progression, lending further credence to the concept that thrombosis generally reflects adverse tumor biology [21].

However, not all papers published to date are unanimous with respect to the impact of VTE/AT on survival in among oncological patients treated with ICI. For example, Moik et al. [12] established that median OS was lower among subjects with VTE (25.5 vs. 11.6 months, *p* < 0.001) although they did not find that AT influenced prognosis. The series by Sussman et al. [17] also detected that ICI-associated thrombosis affected survival, with a median OS for cases with VTE of 20 months, while in those cases that did not develop VTE, the median was not reached. However, works by Deschênes-Simard et al. [16] and Gutierrez-Sainz et al. [18] failed to detect any statistically significant relationship between survival and development of ICI-associated VTE.

Finally, we proceed to address the identification of factors that can predict VTE/AT in subjects treated with ICI. As it pertains to our series, it is worth noting that in both patients with lung cancer and those with melanoma, elevated NLR at the beginning of ICI increases the risk of subsequent VTE/AT. This is not surprising given that studies have already been published about how this parameter is linked to thrombotic burden [22].

In the case of melanoma in particular, it is also interesting to remark on the greater probability of VTE/AT associated with ICI when LDH at the start of this treatment modality is elevated. Elevated LDH negatively affects the prognosis of a patient with melanoma [23] thus, it seems only logical that it should correlate with the risk of VTE/AT when starting ICI, given the impact of this type of event on survival, as evinced by the series studied in this work.

Some of the previously referenced works attempt to describe predictor variables of VTE/AT. Moik et al. [12], Ando et al. [13], and Gong et al. [15] report a history of VTE as being predictive of ICI-associated thrombosis. Our study population bears out this finding. Nevertheless, there are two points that cause the data not to be entirely comparable. The first is that we have found the association when thrombosis occurs in the interval between the diagnosis of cancer and the onset of ICI, while the second is that this association is only apparent in patients with lung cancer. In contrast, it is worth mentioning that Kewan et al. [14] report anemia at the onset of ICI to be a prognostic factor, albeit in our series, this has only been described as being associated with the risk of VTE/AT in patients with lung cancer.

Finally, other factors described in other studies as predictors of VTE/AT in subjects treated with ICI should be highlighted, although no association was found in our series. These variables are high Khorana scores [14, 15, 17], ECOG of < 2 [14], HBP [15], age < 65 years [16] PDL1 > 1% [16], treatment with a combination of two immunotherapy agents [17], history of coronary artery disease [17], anticoagulant therapy at the start of ICI [17] and being female [18].

Despite the weighty data obtained, this study has some limitations. The first limitation is its retrospective nature. However, this weakness is compensated by two strengths that we deem significant: it is a multicenter study (unlike others that have been presented throughout the discussion section) and the large sample size (especially the lung cancer cohort that consisted of 665 cases). The participating centers are from different parts of Spain, so the data enable us to draw conclusions that are representative of the heterogeneity of patients in our country.

Second, the fact that the incidence rates reported may vary as subsequent series are published. Bearing in mind that ICI has become more widespread in recent years, VTE/AT figures may increase due to that this treatment modality is becoming accessible to more and more people. Likewise, the increased survival associated with this type of treatment implies that the periods during which thrombotic risk is high (either due to persistence of an advanced-stage tumor or to longer exposure to ICI) are becoming more prolonged. Consequently, the possibility of developing VTE/AT increases. This could account for the fact that some of the series discussed [16, 18] have failed to detect an association between survival and VTE/AT.

The third, and last limitation that we would like to mention is the lack of homogeneity with respect to other studies in finding predictors of thrombosis in cancer patients who initiate ICI. We were unable to find a justification for this issue. It is conceivable that larger sample sizes and stratified studies based on different tumor pathologies would enable us to obtain more precise data, given that thrombotic risk is not equivalent across all types of cancer.

## Conclusions

ICI increases thrombotic risk in individuals with lung cancer and melanoma. These thrombotic events impact OS in this population.
